# Phage-Induced Expression of CRISPR-Associated Proteins Is Revealed by Shotgun Proteomics in *Streptococcus thermophilus*

**DOI:** 10.1371/journal.pone.0038077

**Published:** 2012-05-30

**Authors:** Jacque C. Young, Brian D. Dill, Chongle Pan, Robert L. Hettich, Jillian F. Banfield, Manesh Shah, Christophe Fremaux, Philippe Horvath, Rodolphe Barrangou, Nathan C. VerBerkmoes

**Affiliations:** 1 Graduate School for Genome Science and Technology, University of Tennessee, Knoxville, Tennessee, United States of America; 2 Chemical Sciences Division, Oak Ridge National Laboratory, Oak Ridge, Tennessee, United States of America; 3 Department of Earth and Planetary Sciences, University of California, Berkeley, California, United States of America; 4 DuPont Nutrition and Health, Dangé-Saint-Romain, France; 5 DuPont Nutrition and Health, Madison, Wisconsin, United States of America; Aarhus University, Denmark

## Abstract

The CRISPR/Cas system, comprised of clustered regularly interspaced short palindromic repeats along with their associated (Cas) proteins, protects bacteria and archaea from viral predation and invading nucleic acids. While the mechanism of action for this acquired immunity is currently under investigation, the response of Cas protein expression to phage infection has yet to be elucidated. In this study, we employed shotgun proteomics to measure the global proteome expression in a model system for studying the CRISPR/Cas response in *S. thermophilus* DGCC7710 infected with phage 2972. Host and viral proteins were simultaneously measured following inoculation at two different multiplicities of infection and across various time points using two-dimensional liquid chromatography tandem mass spectrometry. Thirty-seven out of forty predicted viral proteins were detected, including all proteins of the structural virome and viral effector proteins. In total, 1,013 of 2,079 predicted *S. thermophilus* proteins were detected, facilitating the monitoring of host protein synthesis changes in response to virus infection. Importantly, Cas proteins from all four CRISPR loci in the *S. thermophilus* DGCC7710 genome were detected, including loci previously thought to be inactive. Many Cas proteins were found to be constitutively expressed, but several demonstrated increased abundance following infection, including the signature Cas9 proteins from the CRISPR1 and CRISPR3 loci, which are key players in the interference phase of the CRISPR/Cas response. Altogether, these results provide novel insights into the proteomic response of *S. thermophilus*, specifically CRISPR-associated proteins, upon phage 2972 infection.

## Introduction

Bacteriophages (phages) are abundant and ubiquitous viruses in most natural environments and play an important role in the ecology of their bacterial hosts. In turn, bacteria have evolved various mechanisms to defend themselves against viral predation. One of these strategies involves the CRISPR/Cas system, in which acquired immunity is achieved against invading nucleic acids, providing resistance that can be passed on to future generations [1]–[4]. Clustered regularly interspaced short palindromic repeats (CRISPRs) are loci found in approximately 46% and 87% of bacteria and archaea, respectively [5]. These hypervariable regions consist of a leader sequence followed by an array of direct nucleotide repeats interspersed with non-repetitive DNA regions called spacer sequences. Immediately flanking the CRISPR loci are CRISPR-associated (*cas*) genes [6]–[8]. Host genomes that have acquired spacers homologous to phage sequences are rendered resistant to that particular phage and are thus termed bacteriophage insensitive mutants (BIMs) [1], [9]. The mechanism of action of the CRISPR/Cas system is mediated by small interfering crRNA (CRISPR RNA) molecules [10]–[14] and occurs in two phases: immunization/adaptation, and immunity/interference [4]. Several studies have established that the immunization process, which is based on novel spacer acquisition, and the immunity process, which is based on crRNA interference by seed sequence interactions with target DNA, rely on the Cas protein machinery, although the roles of the various Cas proteins are elusive [10], [15], [16]. The sequence and function variability across the Cas proteins of the three CRISPR/Cas types [17], along with the functional idiosyncrasies of the various core Cas proteins, have compounded the difficulty of Cas proteins characterization.

The link between CRISPR loci and phage-specific acquired immunity was first demonstrated in *Streptococcus thermophilus*, an economically important lactic acid bacterium used as a starter culture in the production of yogurt and various cheeses [1]. In industrial batch cultures, *S. thermophilus* is subject to phage attack, resulting in a negative impact on the fermentation process, and thus vast economic and manufacturing losses. Therefore, many studies have monitored these phages in hopes of developing anti-viral strategies. Numerous *S. thermophilus* phages have been characterized via comparative genomics and transcriptomics, including phage 2972, a virulent *pac*-type phage composed of an isometric capsid and long non-contractile tail [18], [19]. The structural proteins of phage 2972 have been characterized, including the major capsid protein (orf9), two major (orf15 and orf17) and three minor (orf18, orf19, and orf21) tail proteins, the portal protein (orf5), and the receptor binding protein (orf20) [18]. However, the complete proteome of the virus has yet to be elucidated, rendering an incomplete characterization of the functional signature for phage 2972.

The CRISPR content of various microorganisms, including numerous strains of *S. thermophilus*, have been analyzed allowing characterization of novel spacer additions and strain typing based on spacer content and hypervariability. These features reflect biogeography and provide a historical perspective of exposure to foreign genetic elements [20]–[23]. *S. thermophilus* DGCC7710, the strain used in this study, contains four CRISPR loci within its genome [3]. The CRISPR1 and CRISPR3 loci, both type II CRISPR/Cas systems (Nmeni subtype) [6], [17] are known to be active, with the ability to acquire novel spacers in response to phage challenge [3], [9], [22]. CRISPR2 (Type III system, Mtube subtype) and CRISPR4 (Type I system, Ecoli subtype) loci contain three and twelve spacer sequences, respectively. However, new spacer additions have never been observed at CRISPR2 or CRISPR4 loci despite multiple viral challenges.

In this study, we employed shotgun proteomics via 2D-LC MS/MS to measure the global proteomes of *S. thermophilus* DGCC7710 cells upon infection with phage 2972 at two different multiplicities of infection (MOI). Through this study we were able to simultaneously measure bacterial and phage proteins and gain insights into the phage proteins synthesized as well as the global response of the host upon phage infection. In addition, we monitored the Cas protein abundances from all four CRISPR loci in *S. thermophilus* DGCC7710 as a function of time post infection.

## Materials and Methods

### Bacterial Cultures and Phage 2972 Infection

*Streptococcus thermophilus* DGCC7710 and phage 2972 were obtained from DuPont Nutrition and Health (Madison, WI, USA). *S. thermophilus* DGCC7710 was cultivated in M17 medium (Difco, Lawrence, KS, USA) supplemented with 0.5% lactose (LM17) at 42°C. A mid-log phase culture (O.D._600_ = 0.4) was spun down (10,000 *g* for 10 minutes), resuspended in fresh LM17 medium containing 10 mM CaCl_2_, then infected with phage 2972 at an M.O.I. of 0.1 or 1 and incubated at 42°C.

### Cellular and Viral Enriched Fraction Preparation

At times 0, 0.5, 1, 2, 4, and 24 hours post-infection (hpi), 10 ml aliquots were taken and separated into cellular fractions or viral enriched fractions via PEG precipitation (for MOI = 1 only). Cellular fractions were obtained by centrifugation at 10,000 *g* for 10 min at 4°C and retaining the pellets. The supernatant was then PEG-precipitated [24]. Briefly, DNase I and RNase were added at a final concentration of 1 µg/ml and incubated for 30 min at room temperature. 1 M NaCl was added to the supernatant incubated for 1 h on ice, then centrifuged at 10,000 *g* for 10 min at 4°C. Phage particles were precipitated by the addition of PEG8000 (Sigma Aldrich, St. Louis, MO) (10% w/v) for 1 h on ice, then centrifuged at 10,000 g for 10 min at 4°C. The pellets were resuspended in SM buffer [24] and equal volume of chloroform (Sigma Aldrich, St. Louis, MO), then spun down and the aqueous phase recovered.

### Protein Denaturation and Digestion

For cell lysis and protein denaturation, cellular pellets were resuspended in 6 M guanidine HCl (Sigma Aldrich St. Louis, MO), sonicated (Branson Sonifier; 10% amplitude, 10 seconds on/off cycles for 10 min total), and incubated at 60°C for 1 h. Protein concentrations were measured using the Pierce bicinchoninic acid assay (BCA) (Thermo Scientific, Rockford, IL) then disulfide bonds were reduced with 10 mM dithiothreitol. The protein solution was diluted to 1 M guanidine in 50 mM Tris (pH 7.6), 10 mM CaCl_2_, and proteins were enzymatically digested into peptides using sequencing-grade trypsin (Promega, Madison, WI). The peptide solutions were desalted by C18 solid-phase extraction (SepPak, Waters, Milford, MA), solvent exchanged into 0.1% formic acid, concentrated, and passed through a 0.45 µm filter (Millipore, Bedford, MA). Samples were frozen at −80°C until analyzed by 2D-LC-MS/MS.

### Nano 2D-LC-MS/MS Analysis

Peptide mixtures were separated using on-line two-dimensional liquid chromatography with a split phase column containing reverse phase (C18) and strong cation exchange (SCX) materials [25]–[27]. Peptides were eluted from the SCX resin by increasing ammonium acetate salt pulses followed by reverse phase resolution over two hour organic gradients as described previously [28]–[30], ionized via nanospray (200 nl/min) (Proxeon, Cambridge MA), and analyzed using an LTQ XL linear ion trap mass spectrometer (Thermo Fisher Scientific, San Jose, CA). Technical duplicates were run for all samples with 22 hour runs for the cellular fractions and 8 hour runs for the PEG-precipitated fractions. The LTQ was run in data-dependent mode (top 5 most abundant peptides in full MS selected for MS/MS) with dynamic exclusion enabled (repeat count = 1, 60 s exclusion duration). Two microscans were collected in centroid mode for both full and MS/MS scans.

### Database Construction and Analysis

A protein database was generated from the genome sequence of *S. thermophilus* strain DGCC7710 (http://compbio.ornl.gov/CRISPRproteomics/) and phage 2972 (GenBank accession no. AY699705) [18], along with other common contaminants such as trypsin and keratins. MS/MS spectra from all LC-MS/MS runs were searched with the SEQUEST algorithm [31] using the database above, and filtered with DTASelect/Contrast [32] at the peptide level with standard filters [SEQUEST Xcorrs of at least 1.8 (+1), 2.5 (+2), 3.5 (+3), DeltCN>0.08]. Only proteins identified with two fully tryptic peptides were considered for further biological study. Representative runs were calculated to have false positive rates <0.3% at the peptide level using reversed database searching. COG (clusters of orthologous groups) assignments for each protein sequence were performed by running rpsblast against the COG database from NCBI, with an *E*-value threshold of 0.00001, and the top hit used for the assignment [33]. All databases, peptide and protein results, MS/MS spectra, and supplementary tables are archived and made available as open access via (http://compbio.ornl.gov/CRISPRproteomics/) website.

### Statistical Analysis

Spectral counts, values that can be used to approximate relative protein abundances in LC-MS/MS analyses [34], were normalized to account for technical variability among runs by equalizing the total spectral counts of all runs in the time course. First, an average of the total spectral counts of all runs in the time course experiment was calculated. Then, the normalization factor for each run was calculated as the ratio of the average total spectral count and the run’s total spectral count. Finally, protein spectral counts per run were normalized by multiplying the raw spectral counts by the run normalization factor. Normalized spectral counts of proteins were compared between two time points to identify proteins with statistically significant abundance changes. Because spectral counts follow a Poisson distribution [35], [36], spectral counts per protein were compared between two time points using the exact Poisson test. As proteins have two replicate spectral counts at every time point, *p* values were calculated by comparing the two closest replicate spectral counts from two time points to minimize type I errors. Proteins with a *p* value less than 0.05 were considered to have a significant abundance change. Pairwise comparisons were performed between each time point after infection and time zero in the three time courses (Table S2). Comparisons were also performed between a time point early in infection and a time point during peak infection: 0.5 and 1 hpi for MOI = 1, and 1 and 2 hpi for MOI = 0.1 (Table S3).

## Results

### Overall Results

*S. thermophilus* DGCC7710 cultures were infected with phage 2972 at an MOI = 1 or MOI = 0.1, and after 0, 0.5, 1, 2, and 24 hours post infection (hpi), cellular fractions were collected and analyzed via nano-2D-LC MS/MS. Uninfected controls were also analyzed in tandem. In addition, at the higher infection rate (MOI = 1), fractions were enriched for phage 2972 via PEG precipitation of the corresponding cell supernatants collected at each time point. Two technical replicates were run per time point. High reproducibility was shown between the replicates (Figure S1). The overall protein, peptide, and spectral counts for each fraction and time point are summarized in Table 1.

**Table 1:** Number of proteins, peptides, and spectra identified by LC-MS/MS in cellular (MOI = 0.1 and 1) or PEG-enriched viral fractions (MOI = 1) at each time point of infection.

Sample Fraction	Time Point (hpi)	Protein Identifications(Total/Viral)	Peptide Identifications(Total/Viral)	MS/MS Spectra (Total/Viral)
Viral (MOI = 1)	0	2/1	14/2	119/3
Viral (MOI = 1)	0.5	6/4	70/15	143/14
Viral (MOI = 1)	1	84/17	1170/266	3311/726
Viral (MOI = 1)	2	86/16	1217/299	3197/824
Viral (MOI = 1)	4	68/12	845/209	2104/544
Viral (MOI = 1)	24	61/14	611/269	1972/1299
Cellular (MOI = 1)	0	625/0	8151/0	27821/0
Cellular (MOI = 1)	0.5	540/18	6503/138	26438/335
Cellular (MOI = 1)	1	477/32	5046/567	12866/2341
Cellular (MOI = 1)	2	616/35	6662/565	14015/1969
Cellular (MOI = 1)	4	477/29	4314/366	12002/1789
Cellular (MOI = 1)	24	560/31	6599/453	15628/1005
Cellular (MOI = 0.1)	0	650/0	6679/0	27499/0
Cellular (MOI = 0.1)	0.5	693/7	6928/34	30023/61
Cellular (MOI = 0.1)	1	810/26	9618/198	35347/415
Cellular (MOI = 0.1)	2	611/28	5525/178	20147/521
Cellular (MOI = 0.1)	4	732/24	8217/170	28244/388
Cellular (MOI = 0.1)	24	780/27	10185/228	26908/446
Cellular (Uninfected)	0	697/0	7892/0	25345/0
Cellular (Uninfected)	0.5	670/0	7370/0	30168/0
Cellular (Uninfected)	1	698/0	8132/0	30637/0
Cellular (Uninfected)	2	717/0	7898/0	28582/0
Cellular (Uninfected)	4	741/0	8940/0	30421/0
Cellular (Uninfected)	24	706/0	8979/0	34137/0

Proteins, peptides, and spectral values are based on non-redundant identifications.

All numbers are averages of two technical replicate runs.

### Viral Proteome Characterization

**Figure 1:**
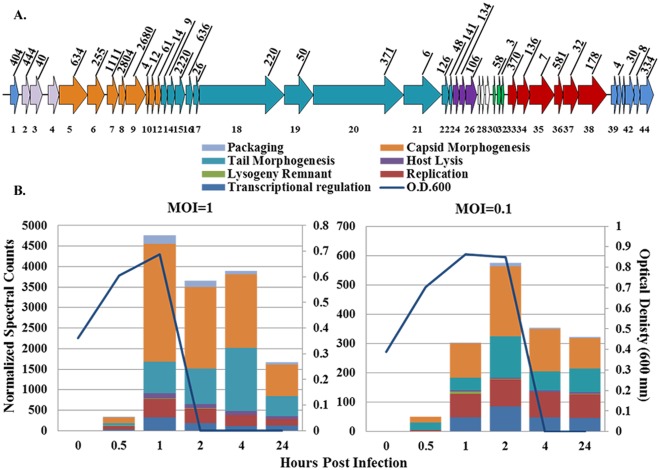
Phage 2972 spectral abundances. A.) Depiction of phage 2972, color coded according to functional modules. Each arrow represents an open reading frame and numbers on top are normalized spectral counts totaled across all MS runs at MOI = 1. B.) Normalized spectral counts were added together at each time point of infection for MOI = 1 (left panel) and MOI = 0.1 (right panel). Optical density measurements (600 nm) (blue line) show cell lysis occurring immediately following the time points in which the highest numbers of phage spectra are detected at each MOI. Colors within each bar correspond to phage functional modules.

The virulent *pac*-type phage 2972 contains 44 open reading frames. Due to two group I introns, the genome encodes 40 putative proteins [18]. In our study, we detected thirty-seven out of the forty predicted proteins, including all of those from the packaging, capsid morphogenesis, tail morphogenesis, and host lysis modules (Figure 1 and Table 2). PEG precipitation was performed on the cultures infected at MOI = 1 in order to enrich the viral structural proteins. However, sequence coverage of the phage structural proteins was, in most cases, better in the cellular fractions than in the virus-enriched fractions (Table 2). In addition, the non-structural proteins were abundantly detected in the whole cell fractions. Therefore, the remainder of the data analyses focused on the cellular fractions.

**Table 2:** Sequence coverages of phage 2972 proteins from virus-enriched and cellular fractions across infection time points.

Module	Orf	Description, Molecular Weight	Virus Enriched Fraction MOI = 1						Cellular FractionMOI = 1						Cellular FractionMOI = 0.1					
			0	0.5	1	2	4	24	0	0.5	1	2	4	24	0	0.5	1	2	4	24
*	1	unknown function, MW:16160								65%	80%	85%	69%	71%			66%	63%	40%	35%
**Packaging**	2	terminase small subunit, MW:16777			19%	24%	25%	56%		31%	85%	71%	67%	81%			19%	38%	25%	11%
	3–4	terminase large subunit, MW: 47066									16%	43%	20%	19%						
**Capsid Morphogenesis**	5	portal protein, MW:57498			50%	58%	50%	51%		33%	56%	58%	55%	53%			43%	25%	24%	25%
	6	head protein, MW:34367			21%	14%	13%	12%		19%	54%	53%	47%	85%			30%	18%	17%	24%
	7	scaffold protein, MW:21262		34%	94%	96%	77%	85%		54%	99%	99%	73%	52%		11%	59%	47%	51%	44%
	8	head protein, MW:12720		41%	96%	96%	98%	100%		86%	88%	91%	100%	89%		50%	77%	66%	63%	55%
	9	major capsid protein, MW:37491	10%	20%	81%	85%	82%	82%		59%	83%	84%	81%	82%		37%	62%	61%	65%	63%
	10	unknown function, MW:5997									89%						53%			
	11	unknown function, MW:13021			31%	61%					50%	47%	54%					37%		
	12	unknown function, MW:11470			66%	73%	68%	70%			58%	64%	39%	58%			35%	30%	30%	45%
**Tail Morphogenesis**	13	unknown function, MW:12495						49%			35%	35%								
	14	unknown function, MW:14637						19%				26%	24%	32%			32%	24%	24%	28%
	15	major tail protein, MW:18525		26%	84%	90%	86%	86%		64%	88%	86%	88%	86%		53%	66%	60%	65%	64%
	16	unknown function, MW:13138								39%	100%	98%	93%	84%		58%	50%	66%	48%	30%
	17	major tail protein, MW:12613									43%	42%	23%	36%						
	18	minor tail protein, MW:153506			9%	12%	8%	17%			14%	20%	14%	14%			2%	8%	5%	10%
	19	minor tail protein, MW:57710			18%	20%	6%	22%			18%	28%	15%	23%				17%	7%	15%
	20	antireceptor, MW:177330			18%	22%	14%	16%		5%	22%	29%	25%	34%			2%	5%	7%	18%
	21	minor tail protein, MW:74279			11%	10%		7%				5%		4%				6%		4%
	22	unknown function, MW:14539			73%	31%					82%	90%	77%	84%				20%	44%	
	23	unknown function, MW:5475			45%						47%	47%	47%	47%		47%				
**Host Lysis**	24	unknown function, MW:12328				33%					33%	38%	33%	38%					28%	23%
	25	holin, MW:12004			31%						75%	74%	72%	59%				39%		
	26	lysin, MW:21754			49%	48%	38%				39%	49%	44%	35%			31%	20%	29%	42%
**Lysogeny Remnant**	30	unknown function, MW:5249																		
	31	cro-like repressor, MW:7850								92%	84%	56%	42%				54%	46%		46%
	32	unknown function, MW:5039								85%							58%			
**Replication**	33	unknown function, MW:18072								53%	65%	64%	52%	60%			32%	32%	43%	52%
	34	unknown function, MW:26164								28%	21%	47%	46%	66%			36%	22%	35%	44%
	35	helicase, MW:50968										9%		6%				10%		9%
	36	unknown function, MW:17290								82%	99%	99%	89%	82%		48%	88%	77%	88%	93%
	37	replication protein, MW:30474									29%	15%	28%	33%			16%			16%
	38	primase, MW:59060								9%	34%	40%	15%	36%			24%	11%	20%	30%
*Transcriptional Regulation	39	unknown function, MW:12133																		
	40	unknown function, MW:9580										25%		37%			52%			25%
	41	unknown function, MW:6311																		
	42	DNA binding protein, MW:19572								23%	41%	18%		52%			54%	54%	48%	57%
	43	unknown function, MW:12132									37%	35%					26%	24%	26%	
	44	unknown function, MW:27652								47%	86%	83%	45%	72%			31%	52%	56%	41%

Values are percent sequence coverages determined by dividing the number of amino acids detected in the mass spectrometry run by the total number of amino acids in a given protein. Numbers are averages between two technical replicates. Phage functional modules are labeled on the right [18]. * Orf1 is part of the transcriptional regulation module. Intron regions are not included in the figure (*orf27–29*) and *orf 3* and *orf4* encode one protein due to a splicing event [18].

The capsid and tail morphogenesis modules encompass all of the structural proteins, most of which are highly represented in our samples. Specifically, capsid morphogenesis proteins account for up to 2,871 normalized spectral counts in one run, and tail morphogenesis proteins account for 1,540 (Table S1 and Figure 1). In turn, individual structural proteins in these modules contribute a high number of total spectra, with up to 2,680 normalized spectral counts for the major capsid protein (*orf 9)*, 2,220 for the major tail protein (*orf 15)*, and 2,804 for one head protein (*orf 8)* across all runs infected at MOI = 1 (Figure 1 and Table S1). In addition, all of the proteins from the host lysis module were synthesized, including a protein of unknown function, the holin, and the lysin *(orfs 24–26)*. The phage proteins that were not detected in our study were genes of unknown function from the transcriptional regulation (*orf 39* & *orf 41*) and lysogeny remnant modules (*orf 30*) (Table 2).

The spectral count abundances of phage 2972 proteins at each time point correlate well with the phase abundance values during the period in which complete cell lysis occurred (Figure 1). Specifically, the highest number of spectra in the MOI = 1 experiment were recorded after one hour and lysis of the cell cultures occurred after two hours. The less robust infection at MOI = 0.1 yielded fewer phage proteins, however the highest number was detected at two hours post-infection, and complete lysis occurred after four hours. The times at which phage protein abundances are highest: 1 and 2 hours post-infection for MOI = 1 and MOI = 0.1, respectively, are defined as the peak infection times in our study (Figure 1).

### *S. thermophilus* DGCC7710 Proteome Characterization

**Figure 2:**
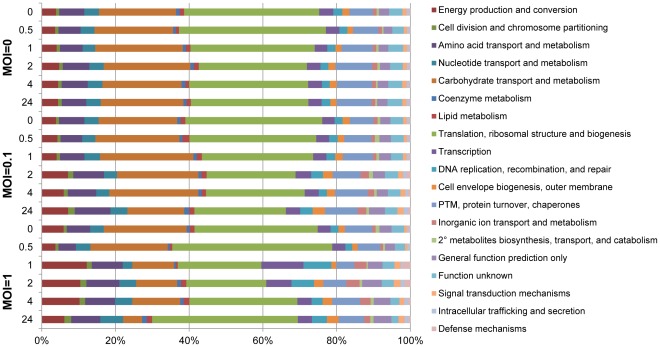
COG classification of *S. thermophilus* proteomes across infection time points. Proteins were grouped into functional categories by COG assignments. Percentages were calculated using normalized spectral counts averaged between two technical replicates.

In total, across all MS runs, 1,013 *S. thermophilus* DGCC7710 proteins were detected (Table S1). As the genome encodes 2,079 open reading frames (http://compbio.ornl.gov/CRISPRproteomics/), this equates to proteomic identification of approximately 50% of the predicted proteins, the highest reported for any lactic acid bacterium to date [37]. A global functional analysis was carried out by grouping host proteins detected at each time point by their COG (clusters of orthologous groups) categories [33] (Figure 2). Host proteins encompassed the range of cellular functions from energy production and conversion to defense mechanisms, with the greatest percentage of proteins in the translation, ribosomal structure and biogenesis, and carbohydrate transport and metabolism categories. The uninfected control cultures did not have any major changes in overall protein functional categories across the six time points measured. However, global changes in the host proteome were detected in phage 2972-infected cultures, including a decrease in protein abundances in the translation, ribosomal structure and biogenesis category around two hours post infection for the lower MOI = 0.1 (37% at time 0 to 24% at 2 hpi), and at one hour post infection for the higher MOI = 1 (33% at 0 hpi to 23% at 1 hpi). These time points correspond to peak infections of the cell populations at each MOI, as described earlier.

**Figure 3:**
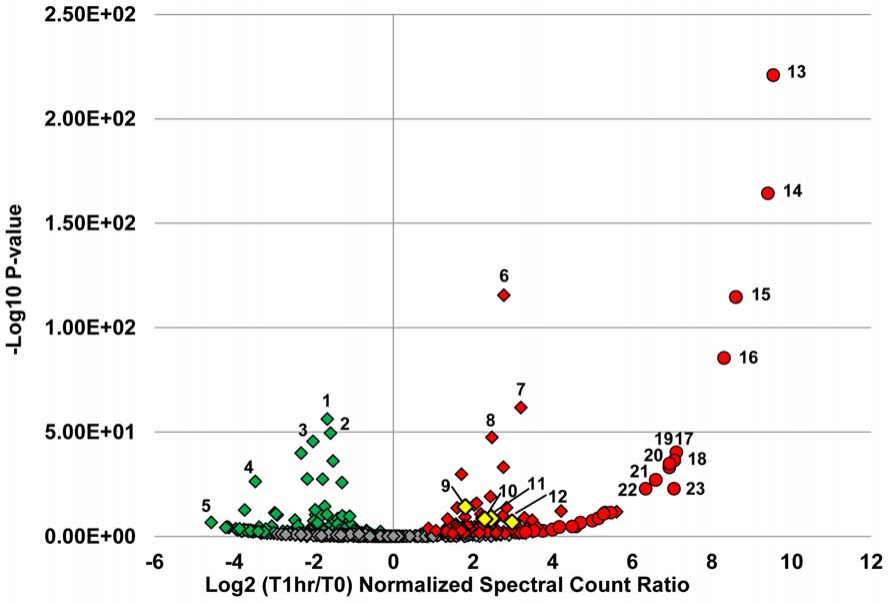
Volcano plot of protein abundance changes during peak infection at MOI = 1. Normalized spectral counts were averaged between two technical replicates and the log_2_ ratios taken between time 0 (pre-infection) and 1 hour post infection (peak infection). P-values were calculated using the exact Poisson test as described in the Materials and Methods section. The -log_10_ of the P-values are plotted on the y-axis. Red color indicates an increase in abundance, green a decrease in abundance, and grey, no change. Diamonds represent host proteins: 1.) glyceraldehyde -3-phosphate dehydrogenase, 2.) pyruvate kinase, 3.) 3-phosphoglycerate kinase, 4.) ribosomal protein S9, 5.) ribosomal protein S8, 6.) ATP synthase, β subunit, 7.) ABC transporter, ATPase, 8.) RNA polymerase, β-subunit. Cas proteins are highlighted in yellow: 9.) Cas6e (CRISPR4), 10.) Cas7 (CRISPR4), 11.) Cas9 (CRISPR1), and 12.) Cas9 (CRISPR3). Phage proteins are depicted in circles: 13. and 14.) head proteins, 15.) scaffold protein, 16.) tail protein, 17.) terminase small subunit, 18.) portal protein, 19.-23.) phage proteins of unknown function.

In addition, at the higher MOI = 1, protein abundances in the carbohydrate transport and metabolism category show a considerable reduction following peak infection (22% at 0 hpi vs. 11% at 1 hpi.) (Figure 2). Decreased abundances of several key enzymes involved in carbohydrate transport and metabolism were detected, including pyruvate kinase, enolase, 6-phosphofructokinase, 3-phosphoglycerate kinase, and glucose-6-phosphate isomerase (Figure 3, Figure S2, and Table S2).

**Figure 4:**
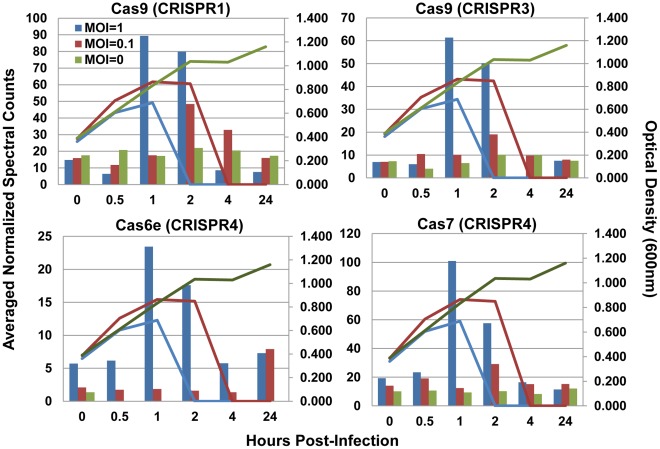
Restriction modification protein subunits increased at peak infection times. Bars indicate normalized spectral counts averaged between two technical replicates and lines are optical density measurements taken at each time point. Untreated cells MOI = 0, green bars and lines, infected cells at MOI = 0, maroon bars and lines, and infected cells at MOI = 1, blue bars and lines. From top left to bottom right: Type I restriction-modification system methyltransferase subunit (ST89_075300), Restriction endonuclease S subunit (ST89_099800) Restriction-modification enzyme type I S subunit; specificity determinant HsdS (ST89_187033), Restriction-modification enzyme type I M subunit; type IC modification subunit HsdM (ST89_187066).

Ribosomal protein abundances decreased during peak infection (41 ribosomal proteins decreased at 1 hpi MOI = 1, 30 decreased at MOI = 0.1) (Figure 3, Figure S2, and Table S2). In contrast, abundances of ABC-type transporter proteins (28 at MOI = 1, 26 at MOI = 0.1), the majority of which are annotated as amino acid transporters but others include oligopeptide, metal ion, and phosphate transporters, increased (Figure 3, Figure S2, and Table S2). The increased expression of ABC transporters is part of the general stress response of these bacteria [38]. Additionally, six subunits of the ATP synthase (α, β, δ, γ, ε,b) were detected and most were up-regulated in response to infection at both MOIs (Figure 3 and Table S2). Interestingly, several restriction-modification protein subunits were also increased at peak infection times including two different methyltransferase subunits (HsdM) and two different endonuclease (HsdS) subunits (Figure 4).

### Analysis of the CRISPR/Cas Response to Phage Infection

**Figure 5:**
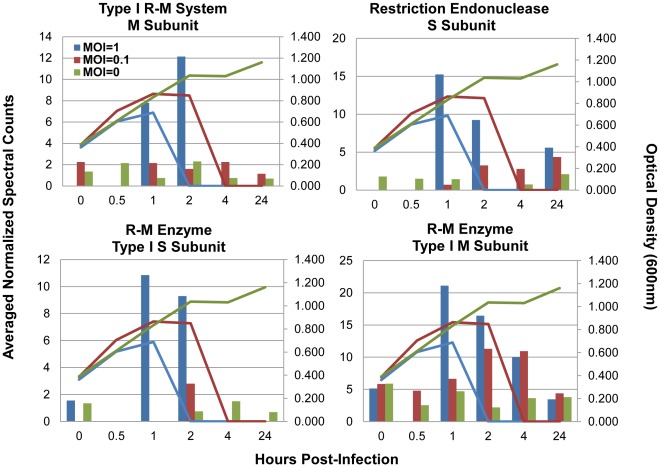
Cas proteins changing in response to phage 2972 infection. Values are normalized spectral counts averaged between two technical replicates. Untreated cells MOI = 0, green bars, infected cells MOI = 0, maroon bars, and infected cells at MOI = 1, blue bars. Lines of the same color represent optical density measurements for each group. From top left to bottom right: Cas9 (ST89_070900) from CRISPR1 locus, Cas9 (ST89_097000) from CRISPR3 locus, Cas6e (ST89_103830) and Cas7 (ST89_103850) from CRISPR4 locus.

The most significant host response to phage 2972 was the increased production of several CRISPR-associated (Cas) proteins. Cas proteins were detected by unique peptides from each of the four loci present in *S. thermophilus* DGCC7710 (Table 3). Some, predominantly from CRISPR2 and CRISPR4, were constitutively expressed throughout the time course, even in the uninfected cells. Interestingly, a clear increase in abundances of several Cas proteins corresponded to peak infections at both MOIs (1 hpi at MOI = 1, 2 hpi at MOI = 0.1) (Figure 5). The most marked increases were seen for the Cas9 proteins from locus CRISPR1 (ST89_070900), and locus CRISPR3 (ST89_147700), and Cas7 from locus CRISPR4 (ST89_103850).

**Table 3:** Expression of Cas proteins from *S. thermophilus* DGCC710 across time.

Protein	Description	Loci	MOI = 1						MOI = 0.1						MOI = 0					
			0	0.5	1	2	4	24	0	0.5	1	2	4	24	0	0.5	1	2	4	24
ST89_070900	Cas9	CRISPR1	15	6	89	80	9	8	16	12	18	48	33	16	18	21	17	22	21	17
ST89_071000	Cas1																			
ST89_071100	Cas2																			
ST89_071200	Cas4																			
ST89_097000	Cas1	CRISPR2									1									
ST89_097100	Cas2																			
ST89_097200	Cas6																			
ST89_097300	Cas10										2							1		1
ST89_097400	Csm2																	1		1
ST89_097500	Csm3		4	3		3		2	4		2	2	1	4	2	4	2	4	4	4
ST89_097600	Csm4									1	1	1		4	1				1	
ST89_097700	Csm5										2		2	2		2	2	1	4	1
ST89_097800	Csm6																			
ST89_147700	Cas9	CRISPR3	7	6	61	50		8	7	10	10	19	10	8	7	4	7	10	10	8
ST89_147600	Cas1																			
ST89_147500	Cas2																			
ST89_147400	Csn2																			
ST89_103880	Cas3	CRISPR4				2				1	1	1		1		1		2		
ST89_103870	Cse1										1		1					1		
ST89_103860	Cse2		1			5		1	1	1	2	1		3			1	1		1
ST89_103850	Cas7		19	23	101	58	16	11	14	19	12	29	15	15	10	11	9	10	8	12
ST89_103840	Cas5					2			3	1	2	4	3	3	5	1	2	3	2	1
ST89_103830	Cas6e		6	6	23	18	6	7	2	2	2	2	1	8	1					
ST89_103820	Cas1					7	2			1										
ST89_103810	Cas2																			

Values are averaged normalized spectral counts taken at each time point from cells infected at MOI = 1, MOI = 0.1, and uninfected cells (MOI = 0). All Cas proteins were detected by unique peptides.

## Discussion

The simultaneous measurement of phage and microbial host proteins over a time course of infection provides opportunity for novel insights into both phage protein production and the host anti-phage response. In this study, we detected nearly all the predicted phage proteins, validating the *in silico* protein predictions. In addition, expression of certain proteins within the cellular fraction and not in the viral enriched fraction suggests that the phage is utilizing the host machinery to produce these proteins, and they are likely not part of the phage structure. This is expected, given that most are encoded by the lysogeny, replication, and transcriptional regulation modules (Table 2). Many virally encoded proteins identified in the cellular fractions were annotated as hypothetical or proteins of unknown function. Although we cannot define their specific functions, their synthesis indicates that they probably play a functional role in phage propagation.

Transcriptomic data for phage 2972 have been reported previously [19]. Transcription of early, middle, and late genes occurs by 27 minutes after infection. However, we focused our analyses around the time of the expected phage burst (40 minutes after exposure) when viral proteins were at abundant levels to allow detection, which required extending the time course past the first infection cycle. Our inferred protein abundances correlate well with transcript abundance patterns, despite the lack of infection synchronicity.

Since we detected the vast majority of host proteins, we were able to characterize the overall host response upon infection with phage. The overall decrease in the translation, ribosomal structure and biogenesis COG category and in ribosomal proteins in particular, at peak infections, reflects the dramatic impact that phage infection has on host physiology, especially immediately before lysis. Some of the changes in host proteome may be the result of phage take-over of cellular processes for transcription and translation of phage material, notably phage DNA packaging and proteins important for particle assembly.

Of particular interest was the detection of Cas proteins throughout our time course. Many Cas proteins were constitutively produced, consistent with reports indicating that crRNA is constitutively transcribed in the host, and can represent the most abundant small RNA species in the cell [39]. Co-constitutive expression of both guide crRNA and Cas proteins would provide the cell with readily accessible defense against invading elements. Given the speed at which viruses can take over the host machinery, and their short replication cycle, constitutive expression of the CRISPR/Cas immune system ensures that the host immune response will be readily available upon infection.

Given that spacer addition has not been detected in CRISPR4 in prior studies [3], [40], it is notable that most of the CRISPR4 Cas proteins were constitutively expressed in uninfected cells, and that some increased in abundance in response to phage exposure. However, it is not known specifically how each locus acts and how the four loci in DGCC7710 interact. The proteins encoded by the CRISPR4 locus are homologous to the Cas proteins of *Escherichia coli* K12, and consist of: Cas1 (endonuclease), Cas2, and Cas3 as well as the Cascade complex (CRISPR associated complex for antiviral defense) [14] which is composed of six copies of Cas7, two copies of Cse2, and one copy each of Cse1, Cas5, and Cas6e [33]. *E. coli* Cas proteins Cse1, Cse2, Cas7, Cas5, and Cas6e are homologous to proteins of the same name in *S. thermophilus* DGCC7710 (ST89_103870, ST89_103860, ST89_103850, ST89_103840, ST89_103830). Cas7 (homologous to Cas7 in *E. coli*, the protein present in the most copies in the Cascade complex) was the most abundant *S. thermophilus* protein and dramatically increased around the time of peak phage 2972 infection. These data suggest that the CRISPR4 locus is functional (though not expanding its spacer inventory).

At peak phage infection, we detected dramatic increase in abundance of Cas9 proteins of CRISPR1 and CRISPR3, the two loci with previously demonstrated CRISPR activity. The Cas9 protein from locus CRISPR1, which is the signature protein for Type II CRISPR/Cas systems, was previously shown to be important in CRISPR-based immunity since deletion of the *cas9* gene (previously called *cas5* or *csn1*) eliminated phage-specific resistance despite the presence of matching spacer sequences [1]. Cas9 was also recently shown to be necessary for the cleavage of invading plasmid and phage DNA [40]. Observing an increase in Cas9 levels at the peak of infection is consistent with a prominent role of Cas9 in CRISPR-encoded immunity [39], [41]. Cas9 proteins contain a HNH-like nuclease motif and are suspected to act on crRNA or foreign nucleic acids, indicating their involvement in the interference phase of the crRNA-mediated response. The increase in critical Cas protein abundance during peak infection indicates that although these proteins are constitutively produced, they can be induced following phage challenge as to increase the level of the primed CRISPR/Cas immune response. This allows the cells to readily acquire novel spacers in response to phage attack, and to mount a Cas9-dependent immune response against invading elements, notably during peak viral infection.

It is important to note that the absence of detection of the other Cas proteins does not necessarily mean a lack of expression. While there are no obvious attributes of the undetected proteins (too small, lacking sufficient tryptic peptides, or too few lysines and arginines) that would prohibit detection by our method, functionally, they may not need to be synthesized at high levels compared with other Cas proteins, and thus may fall below our level of detection. Notably, Cas1, which is found in nearly all genomes containing CRISPR, was not detected in our study. While it is thought that Cas1 plays an important role in the adaptation phase of the CRISPR response [1], [14], [42]–[44], it might only be synthesized by the minority of the cells in the population. In contrast, the Cas9 proteins are more highly detected and are likely expressed by the majority of the cells that take part in the interference phase.

Simultaneously monitoring phage and host protein expression in a population of cells, some of which are developing resistance through the CRISPR/Cas response, required careful consideration of the MOIs and time points used. Historically, low MOIs have been used in the development of bacteriophage resistant mutants (BIMs) through the CRISPR/Cas response, as well as in studies involving phage 2972 [1], [9], [19], [21], [22]. In our study, the timing of such a response is also critical since overwhelming the cells with the lytic phage would cause massive lysis, making it impossible to monitor the development of CRISPR response. Indeed, even at a low MOI, there is already massive lysis. Therefore, the time delay and low MOIs allow us to focus on cells that have survived phage infection, and thus gaze into mounting the CRISPR-mediated phage resistance response.

At the later time points in our time course, even though the majority of the cells are lysed, a subpopulation of resistant cells is still viable (although below the level of detection by optical density), and can regrow when provided fresh media [1], [4], [9], [21]. These bacteriophage insensitive mutants (BIMs) have developed phage resistance via the CRISPR/Cas response. Historically, the percentage of surviving BIM clones that integrate spacers is relatively high (50–90% of clones, depending on challenge conditions) in loci CRISPR1 and CRISPR3 (but not CRISPR2 or CRISPR4), which would explain why the core crRNA containing proteins, Cas7 and Cas9, are upregulated in these loci upon phage challenge. In addition, proteomic analysis of a BIM confirms many of the same Cas proteins are constitutively expressed, including Cas7 and Cas 9, which show comparable abundance levels to uninfected control cells (Table S4).

Recently, transcription profiles of CRISPR systems in *Thermus thermophilus* HB8 upon infection with phage ФYS40 have been reported [45], [46]. *Thermus thermophilus* HB8 contains several CRISPR/Cas systems [47]. In addition to a Type III-B (Cmr) and a Csx, two of these systems, Type I-E (Cse) and Type III-A (Csm), are shared with *S. thermophilus* loci CRISPR4 and CRISPR2, respectively. However, CRISPR1 and CRISPR3, active loci in our model organism, are idiosyncratic type II CRISPR/Cas systems, while those induced by phage in the *Thermus thermophilus* system are type I and type III systems, which have different mechanisms of action.

Typically, in Type I CRISPR/Cas systems the CASCADE complex binds to pre-crRNA, which is then cleaved by Cas6e (in I-E subtypes) or Cas6f (in I-F subtypes) to generate mature crRNAs [13], [16], [17], [42]. Then CASCADE, crRNA, and Cas3 recognize complementary target DNA by sequence-specific hybridization, and cleave it [15]. In contrast, in Type II systems *trans*-encoded small RNAs (tracr RNAs) base pair with repeat regions which are then cleaved by host RNAseII into crRNAs [39]. This requires the aid of Cas9 which then probably targets DNA for cleavage at the protospacer activated motif (PAM) [39], [40]. In type III systems, Cas6 is required for processing crRNA which is transferred to a specific Cas complex (Cmr or Csm) and can target either DNA (III-A) or RNA (III-B) without a the need for a PAM [12], [48].

Additional studies have investigated CRISPR transcription in other systems, including *E. coli* [14], [49], [50], *Sulfolobus* [51] and *P. furiosus* [12]. While transcriptomic studies offer valuable information at the mRNA level, the proteomic approach used in this study is the first to quantify the final protein products, Cas proteins, over a time course of phage infection.

Interestingly, several type I restriction-modification (R-M) protein subunits were also detected during our time course and some increased in abundance at peak infection (Fig 4). Restriction modification systems are a type of anti-viral defense in which invading foreign DNA is cleaved at target sites while host DNA is protected. Type I R-M systems utilize a multifunctional enzyme made up of three subunits encoded by different *hsd* (host specificity determinant) genes. The HsdR (restriction) subunit functions as a restriction endonuclease cleaving foreign DNA while the HsdS (specificity) and HsdM (modification) subunits are sufficient for modification activity and can form an independent methyltransferase (MTase) that specifically recognizes non-palindromic DNA sequences and cleaves at a non-specific site distant from the recognition sequence. Two Type I R-M system methyltransferase subunits (ST89_075300 and ST89_187066) were identified throughout the time course and increased in abundance during peak infection (Figure 4). These two related proteins were distinguishable because they have low amino acid identity and generate unique tryptic peptides upon enzymatic digestion. Similarly, two different type 1 R-M S subunits were identified (ST89_099800 and ST89_187033) and increased in abundance during peak infection. Detection of two distinct M subunits and two distinct S subunits suggests operation of two type I R-M systems.

This study is, to our knowledge, the first to report protein abundance increases of restriction-modification proteins, in direct correlation with time points in which Cas protein abundances are increased. While restriction-modification genes and CRISPR/Cas genes are mutually encoded in lactic acid bacterial genomes [22], [52] it is not clear whether the two anti-viral systems are working simultaneously or if they share components. The expression of proteins from these two systems simultaneously suggests that perhaps there is a connection between R/M systems and CRISPR/Cas systems.

In conclusion, mass spectrometry-based proteomics studies provided insights into the protein profiles of phage 2972 and its host proteome response to viral infection. We showed that, in *S. thermophilus,* the CRISPR/Cas systems are constitutively expressed and can be induced by viral challenge.

## Supporting Information

Figure S1 **Reproducibility between technical replicates.** Normalized spectral counts from two technical replicates plotted against each other, with replicate 1 on the y-axis and replicate 2 on the x-axis. A linear regression was performed, and the slope of the line (m), and R^2^ values calculated providing a statistical measure (a value between zero to one) indicating how well one term predicts another term. All values are >0.93, confirming the technical reproducibility across replicates.
(PDF)

Figure S2 **Color-coded representation of protein abundance changes for all detected proteins across the time courses.** The Poisson exact test was used to determine proteins which were significantly increased or decreased in abundance with respect to time 0. Each line represents a single protein and is colored red if increased, green if decreased, and black if there was no statistically significant change. Proteins are ordered numerically from top to bottom starting with the viral proteins then the host proteins. A list of the proteins along with the p-values is included in table S2.
(PDF)

Table S1 **All proteins detected throughout infection time course.**
(XLSX)

Table S2 **Proteins changing at each time point in relation to time 0 at MOIs = 1, 0.1, and 0.**
(XLSX)

Table S3 **Proteins changing from T0 to early infection, early infection to peak infection, and T0 to peak infection at MOI = 1 and MOI = 0.1.**
(XLSX)

Table S4 **Proteomic Analysis of a Bacteriophage Insensitive Mutant (BIM).** *S. thermophilus* DGCC7710 was infected at an MOI = 0.1 with phage 2972, and mounted a CRISPR response becoming phage resistant. This bacteriophage insensitive mutant, BIM, was co-cultured with the phage for fifty generations, after which the proteome was measured using nano-2D-LC-MS/MS. Results compared with proteome measurements taken before phage inoculation (WT) showed comparable protein, peptide, and spectral identifications (Table S4A). Not surprisingly, the most abundant CRISPR-associated proteins were Cas 9 from CRISPR1 and CRISPR3 and Cas7 from CRISPR4 (Table S4B). However, spectral counts were comparable to those in uninfected cells. Low levels of expression of Cas6e, Cse2, and Csm3 were also detected, which is consistent with our current time course data which shows that many of these proteins are constitutively expressed.
(DOCX)
