# Supplementary figures and images for: Phage-Induced Expression of CRISPR-Associated Proteins Is Revealed by Shotgun Proteomics in Streptococcus thermophilus
Source: PLoS One. 2012 May 30;7(5):e38077. doi: 10.1371/journal.pone.0038077 (PMC3364186; doi:10.1371/journal.pone.0038077)

Figure S1:

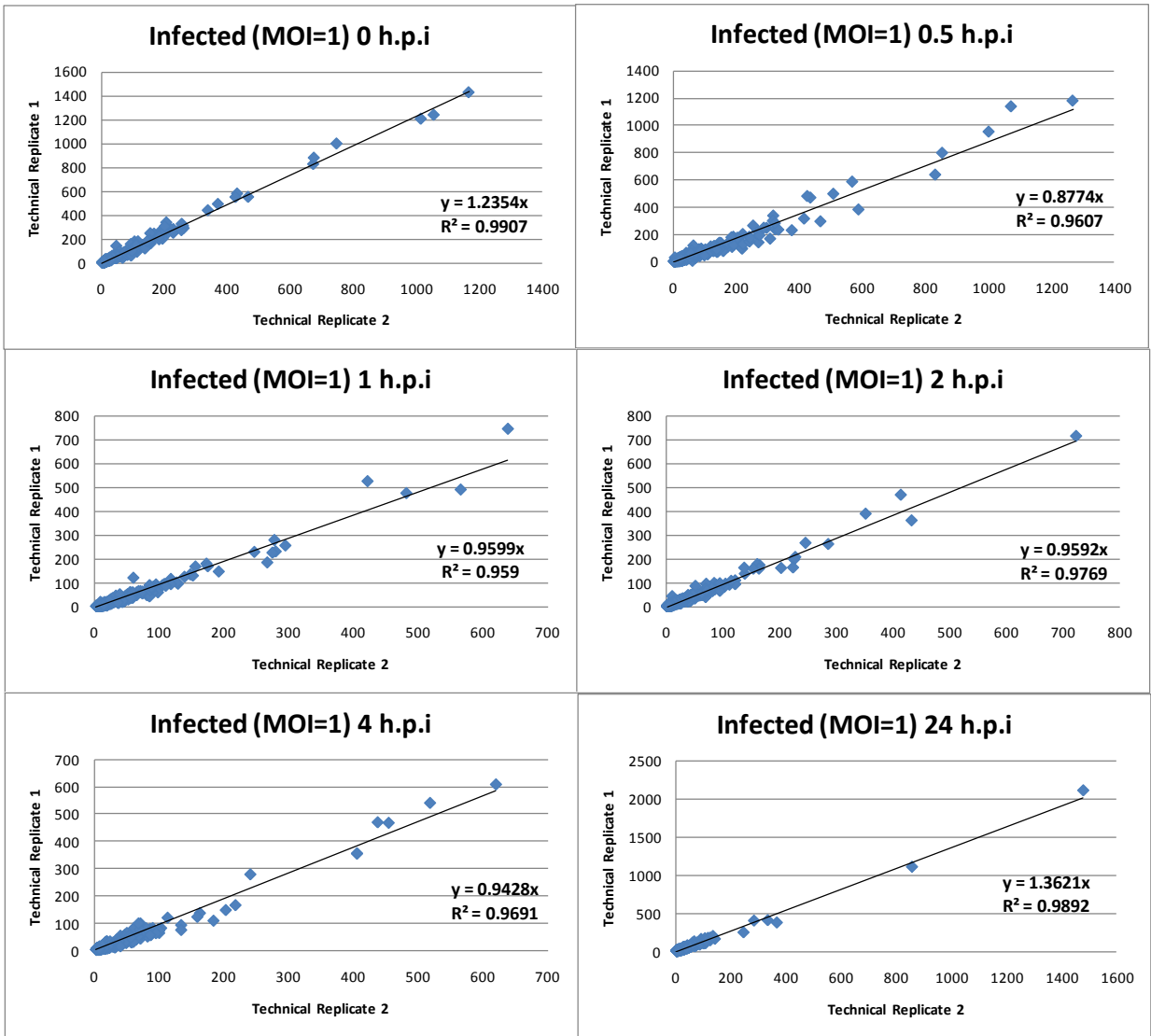

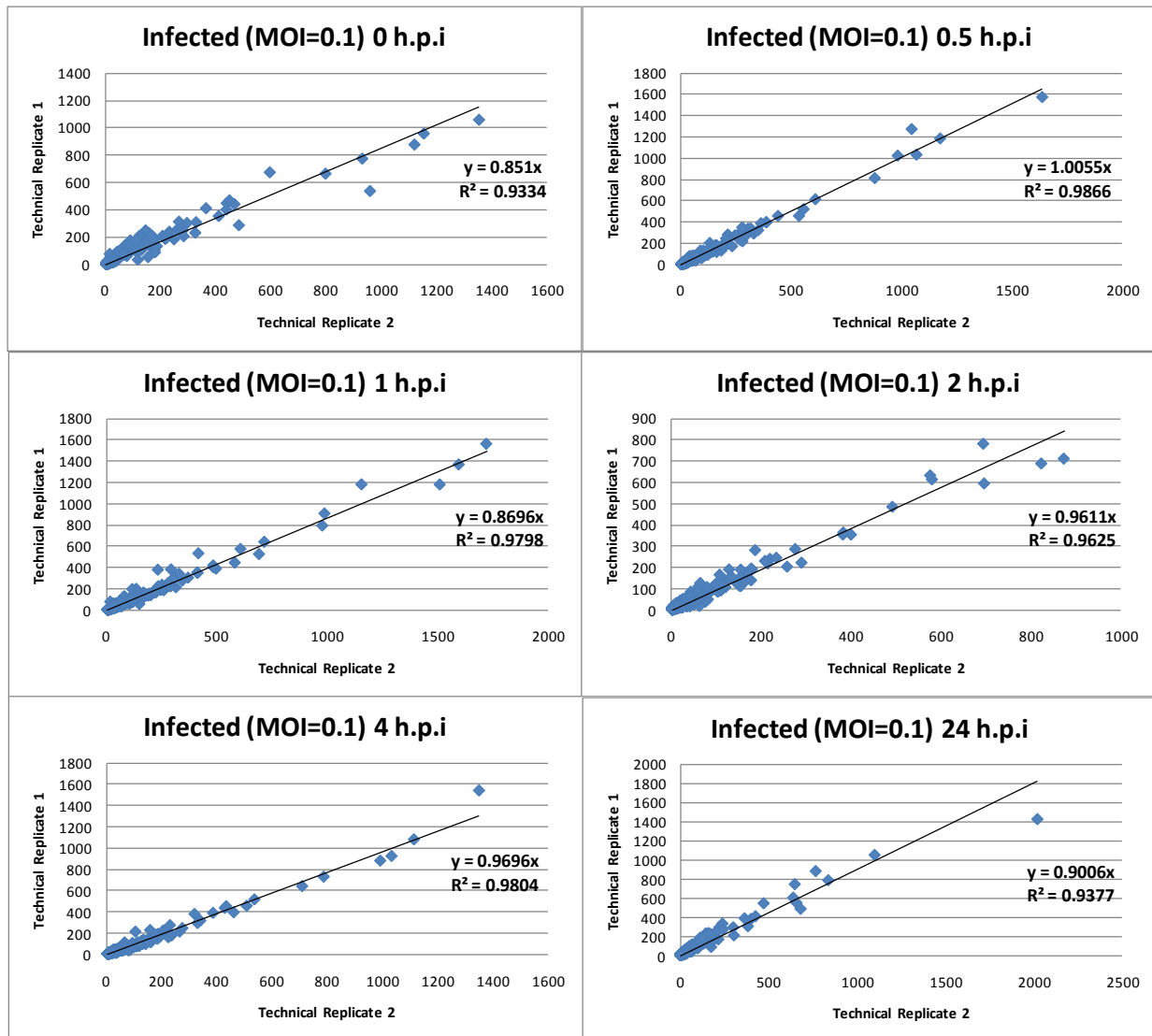

Supplement: Figure S1 — Reproducibility between technical replicates. Normalized spectral counts from two technical replicates plotted against each other, with replicate 1 on the y-axis and replicate 2 on the x-axis. A linear regression was performed, and the slope of the line (m), and R2 values calculated providing a statistical measure (a value between zero to one) indicating how well one term predicts another term. All values are >0.93, confirming the technical reproducibility across replicates. (PDF) [file pone.0038077.s001.pdf]

**Figure S2:**

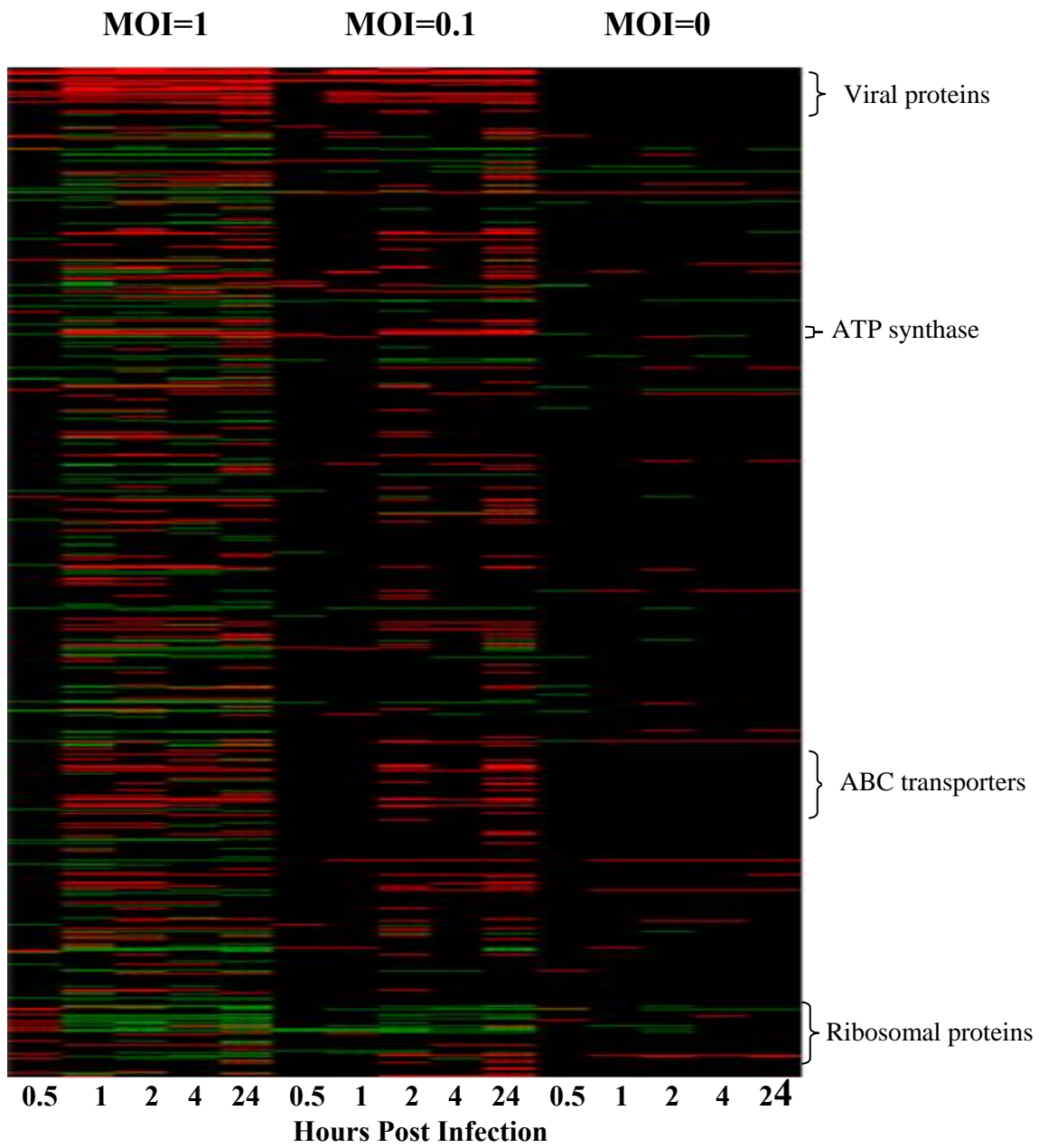

Supplement: Figure S2 — Color-coded representation of protein abundance changes for all detected proteins across the time courses. The Poisson exact test was used to determine proteins which were significantly increased or decreased in abundance with respect to time 0. Each line represents a single protein and is colored red if increased, green if decreased, and black if there was no statistically significant change. Proteins are ordered numerically from top to bottom starting with the viral proteins then the host proteins. A list of the proteins along with the p-values is included in table S2. (PDF) [file pone.0038077.s002.pdf]
