# Supplementary material for: Phage-Induced Expression of CRISPR-Associated Proteins Is Revealed by Shotgun Proteomics in Streptococcus thermophilus
Source: PLoS One. 2012 May 30;7(5):e38077. doi: 10.1371/journal.pone.0038077 (PMC3364186; doi:10.1371/journal.pone.0038077)
Supplement: Table S4 — Proteomic Analysis of a Bacteriophage Insensitive Mutant (BIM). S. thermophilus DGCC7710 was infected at an MOI = 0.1 with phage 2972, and mounted a CRISPR response becoming phage resistant. This bacteriophage insensitive mutant, BIM, was co-cultured with the phage for fifty generations, after which the proteome was measured using nano-2D-LC-MS/MS. Results compared with proteome measurements taken before phage inoculation (WT) showed comparable protein, peptide, and spectral identifications (Table S4A). Not surprisingly, the most abundant CRISPR-associated proteins were Cas 9 from CRISPR1 and CRISPR3 and Cas7 from CRISPR4 (Table S4B). However, spectral counts were comparable to those in uninfected cells. Low levels of expression of Cas6e, Cse2, and Csm3 were also detected, which is consistent with our current time course data which shows that many of these proteins are constitutively expressed. (DOCX) [file pone.0038077.s006.docx]

**Table S4:**

A.) Overview of Proteomic Results from a Phage Resistant *S. thermophilus* Mutant

|  | *Protein*  *Identifications* | *Peptide*  *Identifications* | *MS/MS*  *Spectra* |
| --- | --- | --- | --- |
| WT  (phage sensitive) | 693 | 9057 | 23502 |
| BIM  (phage resistant) | 685 | 9155 | 22849 |

B.) Cas Proteins Detected from a Phage Resistant Mutant

| *Protein* | *Description* | *Loci* | *WT*  *(phage sensitive)* | *BIM*  *(phage resistant)* |
| --- | --- | --- | --- | --- |
| ST89_070900 | Cas9 | CRISPR1 | 21 | 23 |
| ST89_097500 | Csm3 | CRISPR2 |  | 5 |
| ST89_147700 | Cas9 | CRISPR3 | 12 | 10 |
| ST89_103870 | Cse1 | CRISPR4 | 3 |  |
| ST89_103860 | Cse2 | CRISPR4 | 3 | 4 |
| ST89_103850 | Cas7 | CRISPR4 | 20 | 13 |
| ST89_103840 | Cas5 | CRISPR4 | 3 |  |
| ST89_103830 | Cas6e | CRISPR4 | 2 | 5 |
